# Naturally-acquired cellular immune response against *Plasmodium vivax* merozoite surface protein-1 paralog antigen

**DOI:** 10.1186/s12936-015-0681-8

**Published:** 2015-04-15

**Authors:** Siriruk Changrob, Chaniya Leepiyasakulchai, Takafumi Tsuboi, Yang Cheng, Chae Seung Lim, Patchanee Chootong, Eun-Taek Han

**Affiliations:** Department of Clinical Microbiology and Applied Technology, Faculty of Medical Technology, Mahidol University, Bangkok, Thailand; Division of Malaria Research Proteo-Science Center, Ehime University, Matsuyama, Ehime 790-8577 Japan; Department of Medical Environmental Biology and Tropical Medicine, School of Medicine, Kangwon National University, Chuncheon, Gangwon-do 200-701 Republic of Korea; Laboratory of Malaria and Vector Research, National Institute of Allergy and Infectious Diseases (NIAID), National Institutes of Health (NIH), Rockville, MD 20852 USA; Department of Laboratory Medicine, College of Medicine, Korea University Guro Hospital, 97 Guro Dong Gil, Guro Gu, Seoul, 152-703 Republic of Korea

**Keywords:** *Plasmodium vivax*, PvMSP1P, Cellular immune response, Patients, Infection

## Abstract

**Background:**

*Plasmodium vivax* merozoite surface protein-1 paralog (PvMSP1P) is a glycosylphosphatidylinositol-anchored protein expressed on the merozoite surface. This molecule is a target of natural immunity, as high anti-MSP1P-19 antibody levels were detected during *P. vivax* infection and the antibody inhibited PvMSP1P-erythrocyte binding. Recombinant PvMSP1P antigen results in production of a significant Th1 cytokine response in immunized mice. The present study was performed to characterize natural cellular immunity against PvMSP1P-19 and PvDBP region II in acute and recovery *P. vivax* infection.

**Methods:**

Peripheral blood mononuclear cells (PBMCs) from acute and recovery *P. vivax* infection were obtained for lymphocyte proliferation assay upon PvMSP1P-19 and PvDBP region II antigen stimulation. The culture supernatant was examined for the presence of the cytokines IL-2, TNF, IFN-γ and IL-10 by enzyme-linked immunosorbent assay (ELISA). To determine whether Th1 or Th2 have a memory response against PvMSP1P-19 and PvDBPII protein antigen, PBMCs from subjects who had recovered from *P. vivax* infection 8–10 weeks prior to the study were obtained for lymphocyte proliferation assay. Cytokine-producing cells were analysed by flow cytometry.

**Results:**

IL-2 was detected at high levels in lymphocyte cultures from acutely infected *P. vivax* patients upon PvMSP1P-19 stimulation. Analysis of the Th1 or Th2 memory response in PBMC cultures from subjects who had recovered from *P. vivax* infection showed significantly elevated levels of PvMSP1P-19 and PvDBPII-specific IFN-γ-producing cells (*P*  <  0.05). Interestingly, the response of IFN-γ-producing cells in PvMSP1P stimulation was fourfold greater in recovered subjects than that in acute-infection patients. CD4^+^ T cells were the major cell phenotype involved in the response to PvMSP1P-19 and PvDBPII antigen.

**Conclusions:**

PvMSP1P-19 strongly induces a specific cellular immune response for protection against *P. vivax* compared with PvDBPII as the antigen induces activation of IFN-γ-producing effector cells following natural *P. vivax* exposure. Upon stimulation, PvMSP1P-19 has the potential to activate the recall response of Th1 effector memory cells that play a role in killing the parasite.

## Background

*Plasmodium vivax* malaria is the most geographically widespread *Plasmodium* species and the second leading cause of malaria, especially in Southeast Asia and South America [1]. It persists as an important public health problem due to the emergence of chloroquine-resistant *P. vivax* parasites and the occurrence of severe and fatal vivax malaria cases [2,3]. An important part of any control strategy is the implementation of a vaccine capable of inducing protective immunity against *P. vivax*.

Two *P. vivax* vaccine candidates for the exo-erythrocytic stage, *P. vivax* circumsporozoite protein (PvCSP) and *P. vivax* surface protein 25 (Pvs25), have only entered clinical trials, whereas others are undergoing preclinical trials. Blood-stage antigens, such as apical membrane protein 1 (AMA-1), merozoite surface protein 1 (MSP-1) and Duffy binding protein (DBP), are absolutely required for vivax invasion of host erythrocytes. These are responsible for clinical manifestation of vivax disease and target vaccine candidates [4,5]. A blood-stage vaccine would be a valuable tool to reduce both the morbidity and mortality of malaria disease as well as to decrease parasitemia. Several previous reports suggested the feasibility of a blood-stage vaccine. First, immunity against blood-stage antigens can be acquired as a result of natural exposure to *P. vivax* infection. Serological responses to blood-stage antigen and the inhibitory effect of antibodies against erythrocyte binding increase with age, suggesting a boosting effect due to repeated exposure through recurrent infection [6,7]. Second, blood-stage antigen immunisation enhanced protection against *P. vivax* infection [5,8]. These data suggest the immunogenicity of blood-stage antigen. However, the ability of blood-stage antigen to undergo rapid mutation presents an important challenge for vaccine development [9-11]. An effective blood-stage vaccine must generate both humoral and cellular immune responses, overcome genetic restriction, and stimulate memory cells.

Identification of new potential vaccine antigens is important for the development of a successful novel blood-stage vaccine. Data regarding humoral and cellular immune responses against parasite molecules during natural exposure are required at the epidemiological level [12]. T cells are thought to play a central role in the regulation of immune responses and the formation of immunological memory, which can control and eliminate infection. The identification of T-cell epitopes capable of eliciting an immune response in individuals of different genetic backgrounds is necessary for design of a subunit vaccine [13,14]. Studies in both mice and humans have repeatedly shown that proinflammatory cytokines, such as interleukin-12 (IL-12), gamma interferon (IFN-γ) and tumour necrosis factor (TNF) are essential mediators of protective immunity to erythrocytic-stage malaria parasites [15].

With regard to the role of new parasite antigens in the generation of both humoral and cellular responses, in this study, cellular immunity against merozoite surface protein-1 paralog of *P. vivax* (PvMSP1P) was evaluated in *P. vivax* exposed individuals. PvMSP1P is a glycosylphosphatidylinositol (GPI)-anchored blood-stage protein, but is not closely related to merozoite surface protein-1 (MSP1) (11% identity and 22% similarity) [9,16]. The molecular mass, number and location of Cys residues on PvMSP1P are similar to those of PvMSP1, whereas it shows no hyperpolymorphism in the C-terminal sequence. PvMSP1P contains double EGF-like domains that play roles in merozoite invasion. In a previous *in vitro* study, a cytoadherence assay indicated strong binding between pEGFP-PvMSP1P-transfected COS7 cells and human erythrocytes. Serological responses to PvMSP1P-19 antigen and the inhibitory effect of anti-PvMSP1P-19 antibodies were demonstrated in individuals acute *P.* vivax infection [17]. Splenocytes from mice immunized with PvMSP1P-19 showed considerate secretion of Th1 cytokines [18]. These data indicate that PvMSP1P-19 antigen shows high immunogenicity in terms of induction of humoral and cellular immune responses during *P. vivax* infection. However, use of PvMSP1P-19 antigen as a novel vaccine candidate requires an evaluation of the naturally acquired cellular immune response as well as the memory response of T cells. This study was performed to evaluate naturally-acquired cellular immunity against PvMSP1P-19 antigen in subjects exposed to *P. vivax* to determine its ability to stimulate T-cell function and induce a recall response of memory T cells during *P. vivax* infection.

## Methods

### Sample collection

The study was performed in malaria-endemic areas of southern Thailand where both major species of malaria, *P. vivax* and *Plasmodium falciparum*, are common. Peripheral blood samples were obtained at malaria clinics in Tha Sae, Chumphon province, which is in the southern peninsular region of Thailand, from 20 patients with acute *P. vivax* infection and 15 convalescing individuals who had recovered from *P. vivax* infection about 8–10 weeks prior to the study. The patients were from 18–63 years old. Samples from healthy individuals as controls were also collected from 20 malaria-naive volunteers from the Faculty of Medical Technology, Mahidol University, who had no history of exposure to malaria. Venous blood samples (10 mL) were collected by venipuncture in heparin tubes for preparation of plasma and peripheral blood mononuclear cells (PBMCs). Vivax samples were diagnosed from thick and thin peripheral blood smears by Giemsa staining. This study was approved by the Committee on Human Rights Related to Human Experimentation, Mahidol University, and the Ministry of Health, Thailand (MUIRB2012/079.2408).

### Recombinant PvMSP1P-19 protein expression

The gene fragment encoding PvMSP1P-19 was amplified by PCR from genomic DNA of the *P. vivax* Sal I strain sequence, and cloned into the *Xho*I and *Not*I sites of the pEU-E01-His-TEV-MCS vector (CellFree Sciences, Matsuyama, Japan). The inserted nucleotide sequence was confirmed using an ABI PRISM 310 Genetic Analyzer and a BigDye Terminator v1.1 Cycle Sequencing kit (Applied Biosystems, Foster City, CA, USA). Highly purified plasmid DNA is required for *in vitro* transcription and subsequent translation. Plasmid DNA was then prepared using a Maxi Plus™ Ultrapure plasmid extraction system (Viogene, Taipei, Taiwan) according to the manufacturer’s instructions. Purified DNA was eluted in 0.1× TE buffer (10 mM Tris–HCl, pH 8.0, 1 mM EDTA) and used for recombinant protein expression by the wheat germ cell-free (WGCF) system (CellFree Sciences) described previously [19,20]. The recombinant PvMSP1P-19 protein was purified using a Ni-nitrilotriacetic acid agarose column (Qiagen, Valencia, CA, USA). *Plasmodium vivax* Duffy binding protein (PvDBP) region II, the expression vector for which was kindly provided by John H Adams, Department of Global Health, University of South Florida, USA, was expressed as described previously [21,22].

### Lymphocyte proliferation assay

Heparinized whole blood was diluted in incomplete RPMI 1640 medium at a dilution of 1:1. PBMCs were isolated by overlaying with Ficoll-Hypaque (Stemcell Technologies, Vancouver, CA, USA) and centrifuged at 2,000 rpm, 20°C for 30 min, and then washed twice with incomplete RPMI 1640 medium. The PBMCs were then suspended in complete RPMI 1640 medium containing 10% foetal bovine serum at a density of 3 × 10^6^ cells/mL. PBMC viability >  90% was required for lymphocyte proliferation assay (LPA). LPAs were carried out to evaluate cellular immunity against PvMSP1P-19. Aliquots of 2.5 × 10^5^ cells/well of PBMCs were distributed in triplicate in 96-well flat-bottomed tissue culture plates (Corning Inc., New York, NY, USA) in a volume of 100 μL. The cells were then stimulated by adding 100-μL of 10-μg/mL purified recombinant antigen PvMSP1P-19 or PvDBP region II diluted in incomplete RPMI 1640. The complete RPMI 1640 medium and 2% v/v of PHA were used as negative and positive controls, respectively. Cells were cultured for 96 h at 37°C under 5% CO_2_. After 48 and 96 h, the culture supernatant was harvested for cytokine detection.

### Cytokine assay

Cytokine levels in culture supernatant obtained after 48 and 96 h of stimulation with PvMSP1P-19 were measured using an ELISA Cytokine Kit (BD OptEIA™; BD Biosciences, San Diego, CA, USA). Briefly, 96-well ELISA plates were coated with monoclonal antibodies specific to IL-2, IL-10, IFN-γ and TNF. First, 50 μL of ELISA diluent were added to each well, and then 100 μL of standard reagent or culture supernatant were added to duplicate wells. The plates were shaken for 5 s and incubated for 2 h at room temperature. Streptavidin-horseradish peroxidase conjugate mixed with biotinylated anti-human cytokine antibodies was added, followed by incubation for 1 h at room temperature. The plates were washed, followed by addition of 100 μL of chromogenic substrate tetramethybenzidine (TMB) solution to each well. After incubation for 30 min at room temperature, the reaction was stopped with 50 μL of stop solution. The optical density at 450 nm (OD_450_) was measured within 30 min after addition of stop reagent. Each ELISA plate included a human cytokine standard curve, which was used to calculate cytokine concentrations. All specimens were analysed in duplicate and the means of the two values were used in the analyses.

### Evaluation of the response of IFN-γ/IL-10 cytokine-producing cells

To evaluate the response of helper T cells upon PvMSP1P-19 stimulation, the cell phenotypes were analysed by flow cytometry (BD FACScanto II; Becton Dickinson, Oxford, UK). Th1 or Th2 cells were defined by staining with fluorochrome-conjugated monoclonal antibodies specific to CD3 (Alexa700), CD4 (PerCP Cy5.5) and CD8 (APC Cy7) on surface antigen and fluorochrome-conjugated monoclonal antibodies specific to IFN-γ (PE Cy7), IL-2 (APC), IL-4 (Alexa Fluor488) and IL-10 (PE). Briefly, PBMCs were plated in 96-well flat-bottomed tissue culture plates (Corning Inc.) at final concentrations of 5 × 10^5^ – 1 × 10^6^ cells/well in complete RPMI 1640. Cells were stimulated by 20 ng/mL PMA plus 1 μg/mL ionomycin (Sigma, St. Louis, MO, USA) or 10 μg/mL recombinant PvMSP1P-19 antigens. To block cytokine secretion, 10 μg/mL of Brefeldin A (Sigma) was added to the culture medium and incubated at 37°C under 5% CO_2_. After 6 h of stimulation, cells were harvested into 5-mL polystyrene round-bottomed tubes (Corning Inc.) in 50 μL of staining buffer (PBS, 1% BSA, 0.1% NaN_3_) to determine cytokine production by the activated T cell population. First, the cell surface was stained with anti-CD3/CD4/CD8 at 4°C for 15 min and fixed in 0.5% paraformaldehyde solution at 4°C for 20 min. For intracellular cytokine staining, cells were permeabilized using the BD Cytofix/Cytoperm buffer system (BD Biosciences) for 30 min and stained with anti-IFN-γ/anti-IL-2/anti-IL-10 fluorochrome-conjugated monoclonal antibodies for 20 min in the dark. Finally, cells were washed and maintained in staining buffer prior to flow cytometric analysis. Data were analysed using FlowJo version 7.0 (Tree Star Inc., San Carlos, CA, USA).

### Statistical analysis

Data were analysed using the GraphPad Prism software (San Diego, CA, USA), SigmaPlot (Systat Software, San Jose, CA, USA) and Microsoft Excel 2007 (Microsoft, Redmond, WA, USA). One-way ANOVA with Dunnett’s test was used to evaluate the statistical significance of differences between the means of each group. In all analyses, *P*  <  0.05 was deemed to indicate statistical significance.

## Results

### Lymphocytes from acutely infected *P. vivax* patients responding to PvMSP1P-19

**Figure 1:**
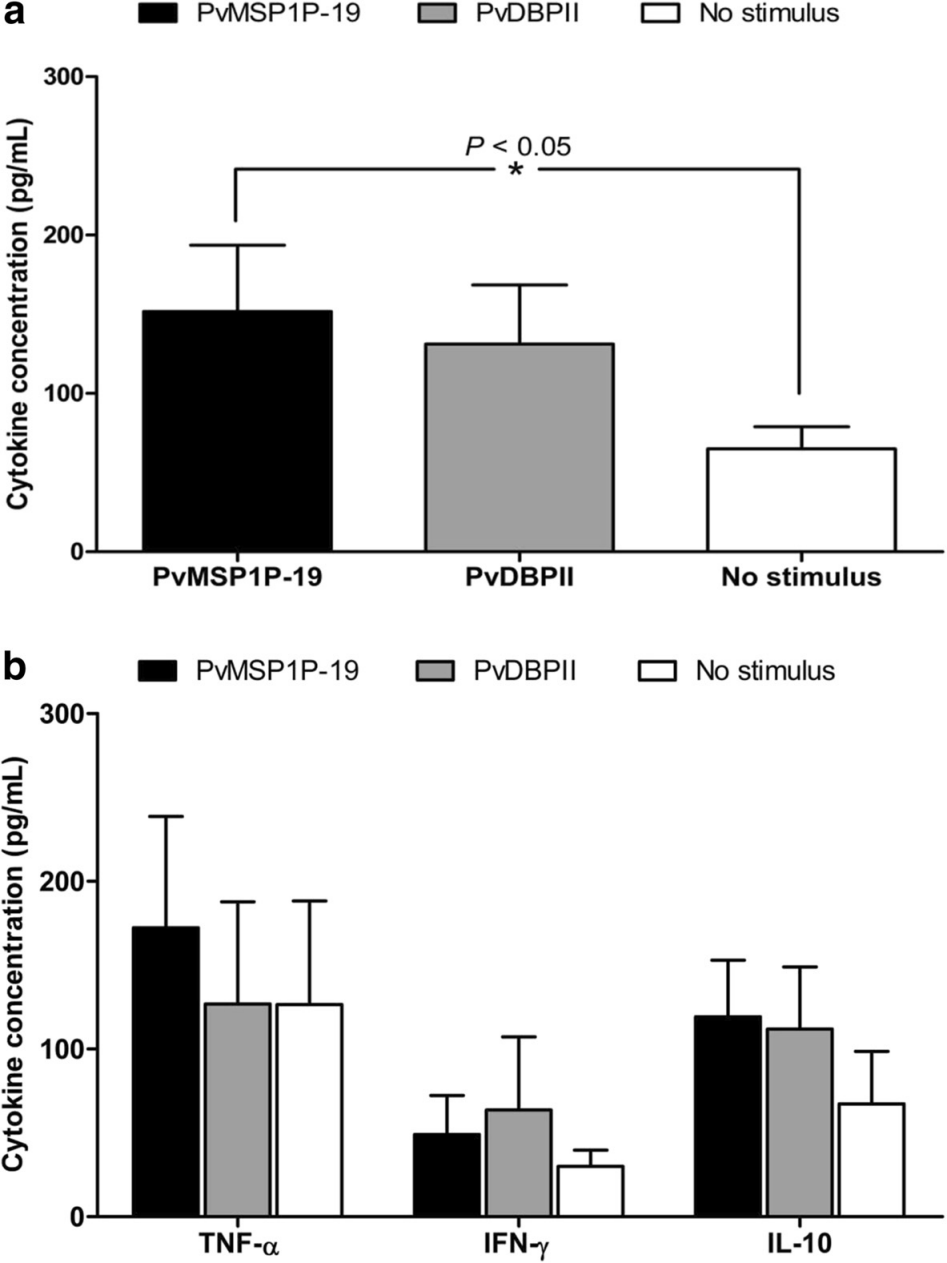
Cytokine production of PvMSP1P-19-stimulated lymphocyte cultures obtained from individuals with acute *P. vivax* infection (*n*  =  15). PBMCs were re-stimulated with PvMSP1P-19 antigen for 48 h and culture supernatant was removed for cytokine detection. **(a)** IL-2 detection after 48 h of *in vitro* stimulation. **(b)** TNF, IFN-γ and IL-10 after 96 h of *in vitro* stimulation.

To evaluate the immunogenicity of PvMSP1P-19 in induction of PvMSP1P-19-specific T cell function during *P. vivax* infection, PBMCs from acutely infected *P. vivax* patients were subjected to lymphocyte proliferation assay. To detect lymphocyte proliferation upon PvMSP1P-19 stimulation, IL-2 levels in the culture supernatant were determined by ELISA. The results showed that PvMSP1P-19 significantly stimulated IL-2 production, indicating that PvMSP1P-19 induces lymphocyte proliferation (PvMSP1P-19  =  151.50  ±  132.96 pg/mL, unstimulated  =  64.88  ±  44.26 pg/mL, *P*  <  0.05, Figure 1a). However, the proinflammatory cytokine, TNF, was detected at low levels in cultures of lymphocytes from acute *P. vivax* patients (PvMSP1P-19  =  172.32  ±  175.86 pg/mL, unstimulated  =  126.64  ±  163.15, *P*  >  0.05).

Since cell-mediated immunity was found to be induced and regulated by cytokines, in this study, the levels of IFN-γ and IL-10 were measured to identify effector T cell phenotype responding to PvMSP1P-19 antigen in acute *P. vivax* infection. PvMSP1P-19 stimulation did not induce a Th1 or Th2 response, as demonstrated by the low levels of IFN-γ (PvMSP1P-19  =  48.88  ±  73.68 pg/mL, unstimulated  =  30.09  ±  30.10 pg/mL, *P* > 0.05, Figure 1b) and IL-10 (PvMSP1P-19  =  119.17  ±  104.10 pg/mL, unstimulated  =  67.32  ±  98.91 pg/mL, *P*  >  0.05, Figure 1b) in PvMSP1P-19 cultures. The levels of all cytokines produced in response to PHA were two- to five-fold higher than those in response to PvMSP1P-19 or PvDBP region II antigen (Figure 1). Lymphocyte from control subjects not exposed to malaria showed no significant response of IL-2, TNF, IFN-γ or IL-10 production upon PvMSP1P-19 stimulation compared to the medium control. These results suggest that *P. vivax* produced PvMSP1P-19-specific effector T cells in response to natural exposure, and a recall proliferative response of effector cells specific to PvMSP1P-19 occurred after restimulation of these effector cells by PvMSP1P-19 antigen.

### Recall response of IFN-γ and IL-10 cytokines against PvMSP1P-19 antigen

**Figure 2:**
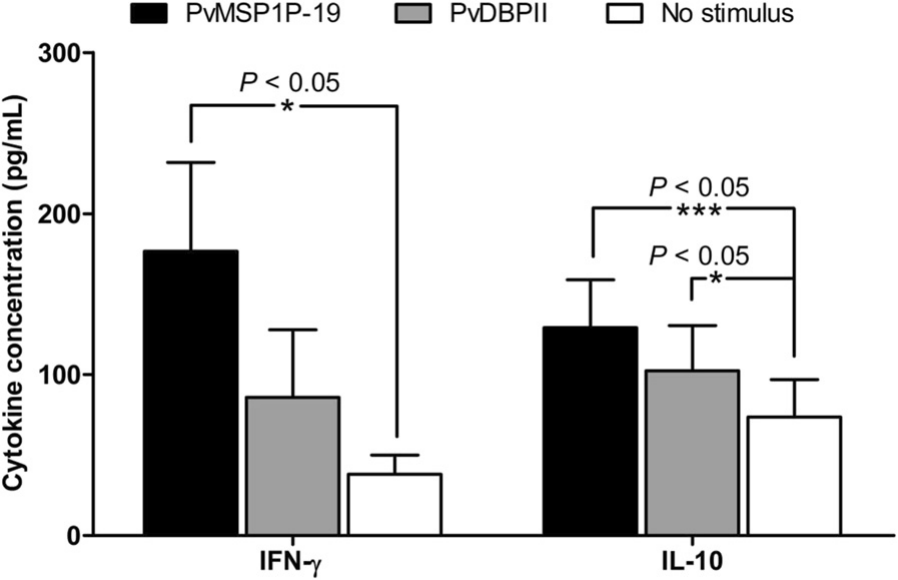
The recall of a cellular immune response specific to PvMSP1P-19 antigen by induction of IFN-γ and IL-10 cytokine responses. PBMCs from individuals who had recovered from *P. vivax* infection 8–10 weeks prior to the study were stimulated by PvMSP1P-19 or PvDBPII antigen with negative (media) and positive controls (PHA) for 96 h. IFN-γ and IL-10 levels in the culture supernatant were measured by ELISA. Data show the average cytokine values from *P. vivax*-recovered subjects (*n*  =  20). Significance was determined by one-way ANOVA with Dunnett’s test. The level of significance was set at *P*  <  0.05.

To evaluate the memory response of lymphocytes on PvMSP1P-19 stimulation, 15 samples of PBMCs were obtained from *P. vivax* individuals who had recovered 8–10 weeks prior to the study for lymphocyte proliferation assay. PvMSP1P-19 significantly stimulated production of IFN-γ by lymphocytes from recovered subjects compared to unstimulated controls (PvMSP1P-19  =  176.58  ±  199.05 pg/mL, unstimulated  =  38.21  ±  43.09 pg/mL, *P*  <  0.05, Figure 2). For IL-10, both PvMSP1P-19 and PvDBP region II antigen strongly induced IL-10 response (PvMSP1P-19  =  113.26  ±  107.90 pg/mL, PvDBPII  =  89.59  ±  99.81 pg/mL, unstimulated  =  65.44  ±  80.50 pg/mL, *P*  <  0.05, Figure 2). The levels of all cytokines produced in response to PHA were fivefold higher than those in response to PvMSP1P-19 antigen. Interestingly, upon PvMSP1P-19 stimulation, the IFN-γ levels in lymphocyte cultures from subjects who had recovered from *P. vivax* infection, were increased by four-folds compared to acute *P. vivax* patients. Moreover, PvMSP1P-19 induced IFN-γ production to a level two-folds, that induced by PvDBP region II (Figure 2). High IFN-γ and IL-10 production upon PvMSP1P-19 stimulation suggested that individuals recovered from *P. vivax* in endemic areas are capable of producing memory IFN-γ and IL-10 cells specific to PvMSP1P-19 antigen. Upon re-exposure to *P. vivax* antigen, PvMSP1P-19 antigen showed the ability to stimulate the memory T-cell response, suggesting that these cells are important regulators of the immune response to *Plasmodium*.

### CD4^+^ T cells play a role in the immune response to PvMSP1P-19 antigen

**Figure 3:**
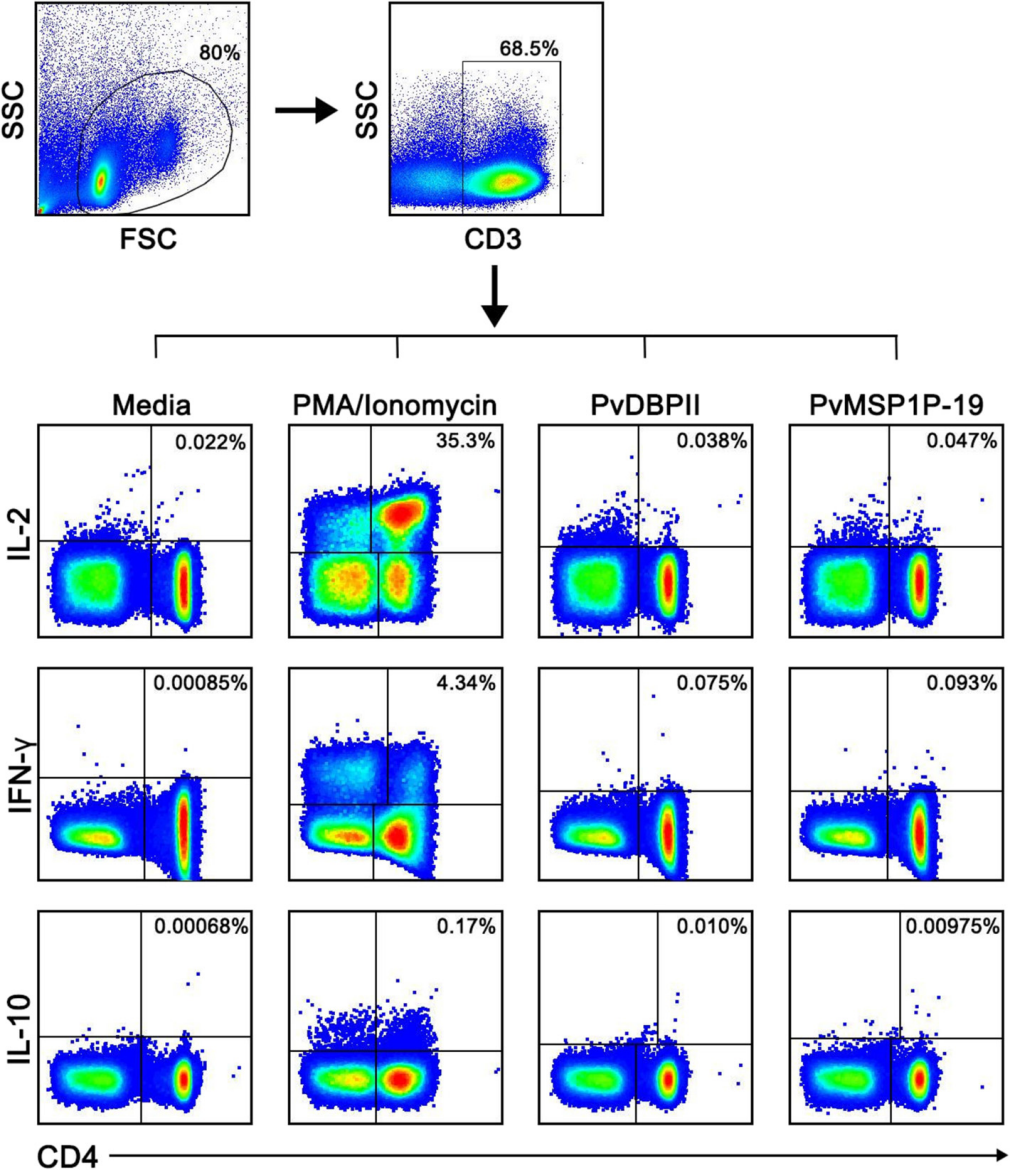
T-cell responses to PvMSP1P-19 antigen using multiparameter flow cytometry. Intracellular cytokine assay demonstrating the T-cell response of *P. vivax*-recovered subjects to PvMSP1P-19 or PvDBPII with negative (media) and positive controls (PMA/Ionomycin). PBMCs from individuals who had recovered from *P. vivax* infection 8–10 weeks prior to the study were removed for lymphocyte proliferation assay and intracellular cytokine detection by flow cytometric analysis. The data are shown as the average levels of cytokine-producing cells in individual subjects (*n*  =  6). This shows the gating strategy to identify IL-2/IFN-γ/IL-10-producing cells.

**Figure 4:**
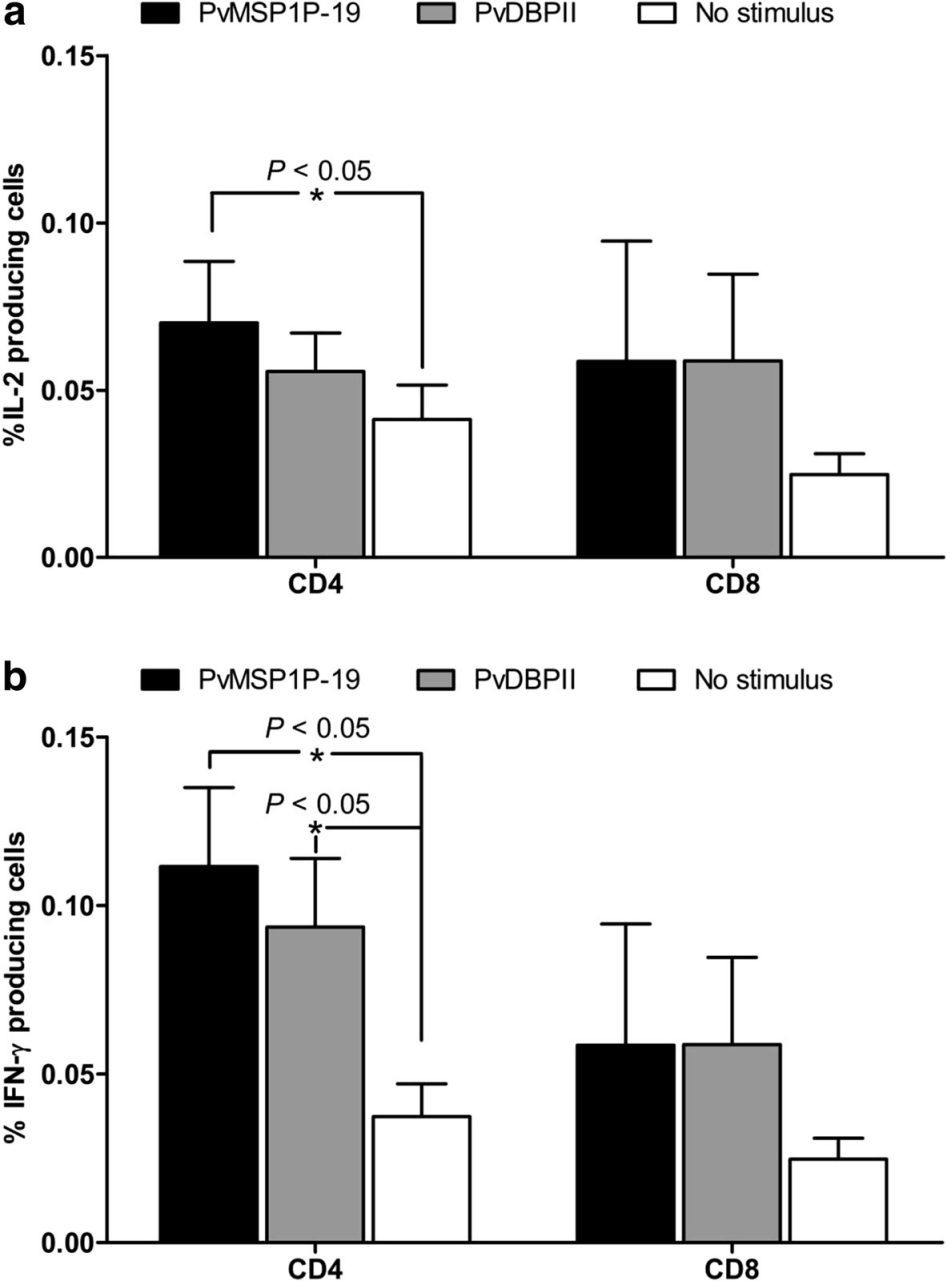
T-cell responses to PvMSP1P-19 antigen using multiparameter flow cytometry. **(a)** Overall PvMSP1P-19-specific IL-2-producing cells. **(b)** IFN-γ-producing cells. Significance was determined by one-way ANOVA with Dunnett’s test. The level of significance was set at *P*  <  0.05.

**Figure 5:**
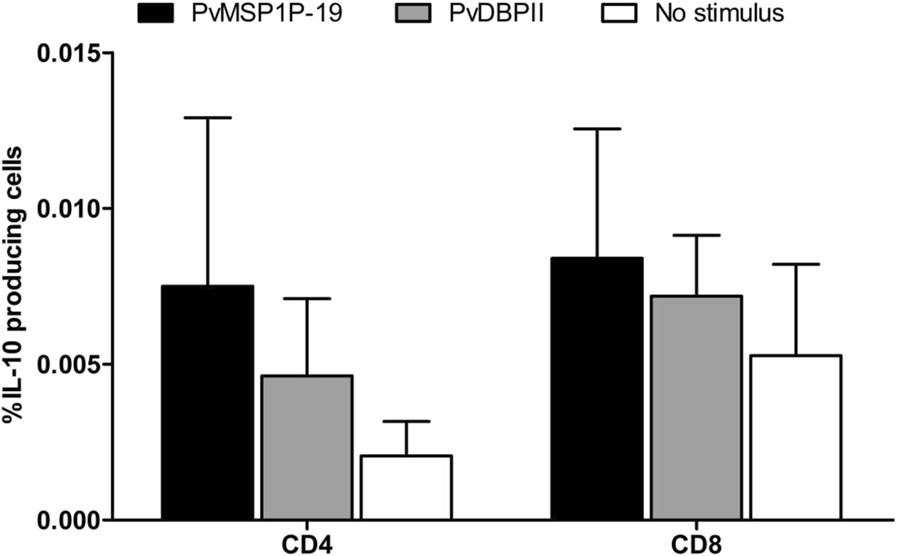
T-cell responses to PvMSP1P-19 antigen using multiparameter flow cytometry. IL-10-producing cells. Significance was determined by one-way ANOVA with Dunnett’s test. The level of significance was set at *P*  <  0.05.

Many different IFN-γ-producing cell subsets, including to αβ T cells, γδ T cells and NK cells, have been shown to be capable of responding to *Plasmodium* parasites. Here, the phenotype of cells involved in the IFN-γ and IL-10 response upon PvMSP1P-19 stimulation was evaluated by flow cytometric analysis (Figure 3). Upon PvMSP1P-19 stimulation, CD4^+^ T cells were the major source of IL-2 and IFN-γ production. PvMSP1P-19 stimulation showed significantly higher IL-2 and IFN-γ levels than the medium control (IL-2, PvMSP1P-19  =  0.070%  ±  0.041%, unstimulated  =  0.041%  ±  0.023%, *P*  <  0.05; IFN-γ, PvMSP1P-19  =  0.112%  ±  0.053%, unstimulated  =  0.037%  ±  0.022%, *P*  <  0.05, Figures 4a-b). The effector CD4^+^ IFN-γ^+^ response to PvMSP1P-19 antigen was double that of CD8^+^ T cells (Figure 4a). Similarly, PvDBP region II antigen also had a high potential to induce CD4^+^ IFN^+^ T cell response (PvDBPII  =  0.094%  ±  0.046%, unstimulated  =  0.037%  ±  0.022%, *P*  <  0.05, Figure 4b). Phenotyping of IL-10-producing cells upon PvMSP1P-19 stimulation showed that CD4^+^ and CD8^+^ T cells were not major sources of IL-10 (CD4^+^, PvMSP1P-19  =  0.008%  ±  0.012%, unstimulated  =  0.002%  ±  0.002%, CD8^+^, PvMSP1P-19  =  0.008%  ±  0.009%, unstimulated  =  0.005%  ±  0.007%, *P*  >  0.05, Figure 5). These data suggest that CD4^+^ T cells dominate over CD8^+^ T cells in the responses against PvMSP1P-19 and PvDBP region II antigens.

## Discussion

Here, the immunogenicity of PvMSP1P-19 in terms of stimulation of cellular immunity was evaluated in subjects exposed to *P. vivax* both during acute infection and 8–10 weeks following recovery. PvMSP1P-19-specific effector T cells from acute and recovery *P. vivax*-exposed subjects produced IL-2 and IFN-γ after re-stimulation with PvMSP1P-19 *in vitro*. CD4^+^ T cells play a major role in the immune response against this antigen. The results of the present study suggest that the immune response of PvMSP1P-19-specific T cells is not only readily induced following *P. vivax* infection but also persists in the absence of further exposure.

In this study, the ability of PBMCs to produce both pro- and anti-inflammatory cytokines in response to stimulation with blood parasite antigen, recombinant PvMSP1P-19 and PvDBP region II antigen was evaluated. PBMCs from symptomatic *P. vivax* infection individuals were obtained in this study as representative of the total malaria-reactive T-cell pool during *P. vivax* exposure. Although malaria-reactive T cells tend to disappear from the peripheral circulation during acute infection, probably migrating to the spleen and liver, they are released back into the periphery upon resolution of the infection. Thus, the selection of peripheral blood as the source of leukocytes has been validated in many previous studies [23].

The parasite induces a specific immune response by stimulating the release of cytokines, and this may have an important function in activating the host’s immune cells to react to the parasite [24,25]. PBMCs from subjects with acute *P. vivax* infection produced high levels of IL-2 but only low levels of IFN-γ and IL-10 were detected in response to PvMSP1P-19 antigen, suggesting that IFN-γ- and IL-10-producing effector cells specific to PvMSP1P-19 did not play a major role in killing the parasite in acute *P. vivax* infection. A significant IL-2 cytokine response may assist IL-4/IL-5/IL-13- or IL-17-producing effector helper cells, as previously shown in studies of cell expansion in acute malaria [26,27]. Macrophages in acutely infected PBMC cultures did not produce TNF. Thus, PvMSP1P-19 protein may not directly induce an inflammatory response, as shown in a previous study in which malarial pigment and certain glycolipids, such as the GPI moiety, stimulated production of the inflammatory cytokine TNF [24].

Immune responses to malaria parasites have been shown to be short-lived following exposure. The response was based mainly on cellular immunity against individual malaria antigens, and was relatively short-lived, declining within a few years of exposure [28] or at least being unstable, but occasionally persisting. However, our understanding of the memory immune response in malaria remains relatively poor [29]. The results of the present study demonstrated a recall response of memory CD4^+^ T cells in natural *P. vivax* exposure. The subsequent cellular immune response in subjects who had recovered from *P. vivax* infection 8–10 weeks previously showed significantly elevated IFN-γ responses to specific PvMSP1P-19 stimulation. CD4^+^ T cells were the main source of IFN-γ in response to this antigen. This was consistent with previous reports that IFN-γ responses are associated with protection against malaria both among volunteers undergoing experimentally induced infection and naturally exposed human populations [28,30-33]. Therefore, elevation of IFN-γ production upon PvMSP1P-19 stimulation could be explained by production of effector Th1 cells or memory cells specific to PvMSP1P-19 in malaria-recovered human populations, and that effector cells could be re-stimulated to produce IFN-γ, indicating a boosting of cellular immunity in individuals following natural exposure to the *P. vivax* parasite. However, further studies are required to determine the longevity of PvMSP1P-19-specific Th1 cells and their protective effects against malaria.

Interestingly, this study showed that no evidence of immediate CD4^+^ and CD8^+^ effector cell producing IL-10 upon short-term *in vitro* PvMSP1P-19 stimulation. IL-10 secretion seemed to occur significant later (accumulating at 96 h in culture supernatant). Levels of IL-10 cytokine significantly increased in lymphocyte cultures from subjects who had recovered from *P. vivax* infection. These data indicated that regulatory responses by IL-10 producing cells may reside within the memory population and that PvMSP1P-19 re-stimulation may be secondary activation of Th1 cells. The cellular source of IL-10 in culture supernatants is not known. There are many sources of IL-10, being produced mainly by Foxp3^+^ regulatory T cells [34]. B cells secreting IL-10 in an antigen specific manner have been described in mice but the antigen specificity of IL-10-secreting B cells is poorly documented in humans and they are found at much lower frequencies than IL-10 producing T cells [35]. Elevation of IL-10 levels after PvMSP1P-19 stimulation *in vitro* supported the sustainability of IL-10-producing effector cells after parasite clearance, as shown previously [30]. A balance of pro- and anti-inflammatory cytokines is required for parasite clearance without inducing excessive host pathology. High levels of IFN-γ production consistent with the IL-10 response upon PvMSP1P-19 stimulation may suggest regulation of the inflammatory response and clearance of the parasite simultaneously.

A number of blood-stage candidate vaccines have progressed to clinical trials but none has yet produced good evidence of protection against clinical malaria. Three of these vaccine candidates, MSP, AMA1 and DBP, were designed to induce protective antibodies capable of reducing or blocking parasite growth [36-39]. Here, the response of T cells against the merozoite antigen, PvMSP1P-19, in comparison to PvDBP region II was demonstrated. PvMSP1P-19 blood-stage antigens stimulated CD4^+^ T-cell response, and as a result showed significantly higher IFN-γ levels in lymphocyte cultures. The activation of CD4^+^ T cells specific to PvMSP1P-19 suggests that this antigen can polarize the Th1 cell response, as shown for MSP1 and MSP3 antigens [39,40], inducing IFN-γ after re-stimulation *in vitro*. The results of the present study support a role for MSP1P-19 protein in the development of IFN-γ-secreting T lymphocytes.

### Conclusions

PBMCs from subjects who had recovered from *P. vivax* infection produced high levels of IFN-γ after PvMSP1P-19 re*-*stimulation *in vitro*. This study suggested that an immune response consisting of PvMSP1P-19-specific T cells is induced following infection and had a boosting effect on cellular immunity in individuals following exposure to the *P. vivax* parasite. The development of a protective *P. vivax* vaccine will require antigen to generate an effector Th1 cell response as well as the ability to induce memory cells, such as the specific immune response against PvMSP1P-19 antigen indicated in this study.

## Abbreviations

PvMSP1P: *Plasmodium vivax* merozoite surface protein-1 paralog
PBMCs: Peripheral blood mononuclear cells
ELISA: Enzyme-linked immunosorbent assay
LPA: Lymphocyte proliferation assay
PHA: Phytohaemagglutinin
